# Efficacy of intranasal ketamine and midazolam for pediatric sedation: A double-blind, randomized clinical trial

**DOI:** 10.22088/cjim.12.4.539

**Published:** 2021

**Authors:** Hossein Khoshrang, Cyrus Emir Alavi, Siamak Rimaz, Ali Mirmansouri, Farnoush Farzi, Gelareh Biazar, Zahra Atrkarroushan, Nazanin Sabet Khadem

**Affiliations:** 1 Anesthesiology Research Center, Department of Anesthesiology, Alzahra Hospital, Guilan University of Medical Sciences , Rasht, Iran; 2 Department of Statistic, Guilan University of Medical Sciences, Rasht, Iran; 3 Student Research Committee, Guilan University of Medical Sciences, Rasht, Iran

**Keywords:** Ketamine, Midazolam, Intranasal, Pediatrics, Sedation

## Abstract

**Background:**

Pediatric patients feel significant fear and anxiety when undergoing surgeries. The ideal drug and its administration route have not been found yet. The aim of this study was to compare the efficacy and safety of intranasal (IN) ketamine and midazolam as premedication in children.

**Methods:**

We studied 71 eligible pediatric patients undergoing elective urologic surgeries, aged 2 to 6 years. The degree of sedation and separation scores was compared between the two groups. Additionally, hemodynamic parameters, before premedication, after induction of anesthesia, and during surgery were documented and compared between two groups. Postoperatively, any side effect was recorded as well.

**Results:**

Finally, the data from 71 children were analyzed. Recovery time was significantly longer in group K (ketamine) compared to group M (midazolam); 27.86±4.42 vs 38.19± 6.67 minutes respectively (P=0.01). No significant difference was observed in terms of sedation score between two groups of K & M; 3.29±0.78 vs 3 ±0.71 respectively (P=0.17), and not regarding separation score; 2.51±0.61 & 2.31±0.52 respectively (P=0.01). Vital signs were kept within the physiological limits in both groups with no marked fluctuations.

**Conclusion:**

To produce sedation in young children, both midazolam and ketamine were effective and safe by IN route.

Premedication in young children for diagnostic or treatment interventions presents challenging conditions. Pediatric patients are not able to receive any explanation about the necessity of being faced with these stressful conditions (1). Separating from parents, operating room, injection, and not familiar environment induce a fearful and traumatic experience which could affect their whole life. A proper drug should be pain-free, effective, safe, rapid onset with a limited duration of action (2). A variety of sedative and analgesic drugs including sufentanyl, dexmedetomidine, midazolam, and ketamine have been administered intra nasally. Studies also indicate that IN rout, is noninvasive, practical with rich vascular plexus cavity which leads to easily achievement of therapeutic drug levels (3). This approach also provides more comfortably compared to the intravenous route (4). The other routes have been tried, but with known disadvantages, such as painful injection in the intramuscular route, delayed recovery (oral), and slow onset with oral or rectal (5). Furthermore, a range of 50- 83% has been described for the bioavailability of IN route of these drugs (6).

Studies have shown that premedication with both IN midazolam and ketamine provides safe conditions for preschool pediatric patients. On the whole, it is well-known that pediatric patients need premedication before separating them from their parents. However, there has not been an agreement about the choice of drug and the ideal route of administration (4, 7, 8). Due to the importance of the issue and the lack of enough knowledge the present research was planned. The purpose of this research was to compare the sedation level, hemodynamic changes, and complications of IN ketamine and midazolam as premedication in pediatric patients' candidate for elective urologic surgeries.

## Methods

 This clinical trial was conducted at Razi hospital, an academic tertiary center affiliated to Guilan University of Medical Sciences (GUMS) during 2017. The study protocol was approved by the Research Ethics Committee of the GUMS (Ref: IR.GUMS.RECs.1396.243) and also was registered as IRCT2016082411766N4. All parents of the enrolled children gave written informed consent before any intervention. After approval by the ethics committee, informed parental consent was obtained.

**Inclusion criteria:** Children aged between 2 and 6 years, weight 10- 20 kg, American Society of Anesthesiology (ASA) class I& II (9) candidate for urologic elective surgeries. 

**Exclusion criteria:** Emergency surgeries, any allergy or contraindication for study drugs, surgery duration less than one hour or more than three hours. The history of hepatic or renal diseases.

Eligible children were randomly allocated to each group of ketamine (K) (manufacturer: laboratoires Sterop, 500 mg/10 ml, made in Belgium) or midazolam (M) (manufacturer: Tehran shimi, 5 mg/1 ml, made in Iran). In group K, 5 mg/kg ketamine and in group M 0.5 mg/kg midazolam were administered slowly drop by drop equally in two nostrils, 30 minutes before anesthesia induction. Each child was evaluated for the degree of sedation according to a five-point scale. 1. Agitated: Patient clinging to parents and/or crying. 2. Alert: The patient is aware but not clinging to parent, may whimper but not cry.3. Calm: Sitting or lying comfortably with spontaneous eye opening.4. Drowsy: Sitting or lying comfortably with eyes closed, but responding to minor stimulation.5. Asleep: Eyes closed, arousable but does not respond to minor stimulation (10, 11). 

The separation score was calculated based on a four-point scale (12). 1-Poor (crying, clinging) 2-Fair (crying but not clinging) 3-Good (whimpers, easily reassured) 4-Excellent (easy separation). 

The child and the physician who administered the drugs and evaluated the cases were blind to the treatment groups. Standard monitoring including respiratory rate, oxygen saturation, end tidal CO2, and noninvasive blood pressure was performed for all children and they underwent general anesthesia in the same manner. Although we were aware of the general anesthesia-related neurotoxicity in young children, performing adequate anesthesia and analgesia with excepted drugs including α2 agonists and opioids were not possible (13-15). 

After intravenous atropine 0.02 mg/kg, propofol 1.5 mg/kg was used to induction of anesthesia. The child was intubated following injection of atracurium 0.5 mg/ kg and anesthesia was maintained with isoflourane and N2O. At the end of the surgery, the effects of muscle relaxants were reversed by atropine 0.02 mg/kg and neostigmine 0.04 mg/kg. The child was transferred to the recovery ward and any complication such as unstable vital signs, secretions, nausea, and vomiting, as well as restlessness and emergence reactions were noted. Finally, the data were analyzed using statistical package for social sciences (SPSS) Version 16 software.

## Results

 Finally, the data from 71 children were analyzed. A total of 36 (50.7%) children were in group K while 35(49.3%) children were in group M. The mean age of our cases was 3.93±1.76 years. The demographic data of the cases are shown in table-1. Recovery time was significantly longer in group K compared to M; 27.86±4.42 & 38.19±6.67 years respectively (P=0.01). No significant difference was observed regarding sedation score between two groups; 3.29±0.78 & 3±0.71, respectively (P=0.17), and not regarding separation score; 2.51±0.61 & 2.31±0.52, respectively (P=0.01) (table 2). The *heart rate* for the group M and K were 126.20±8.76 minutes and 124.23±11.13 minutes before premedication (P=0.13), 127.03±9.45 minutes and 127.81±10.67 minutes after premedication (P=0.54), 115.63±7.73 and 118.81±8.81 minutes preoperatively, respectively (P=0.28). The systolic blood pressure for the group M and K were 97.49±9.13 mmHg and 102.08±11.34 mmHg before premedication (P=0.72), 97.31±9.05 and 104.22±10.46 mmHg after premedication (P=0.51), 88.74±8.42 and 97.11±10.50 mmHg preoperatively respectively (P=0.24). The *diastolic blood pressure* for the group M and K were 63.31±6.51 and 67.75±7.92 mmHg before premedication (P=0.72), 63±6.51 and 68.69±8.04 mmHg after premedication (P=0.51), 57.31±4.02 and 63.11±7.51 mmHg preoperatively, respectively (P=0.24).* SaO2* for the group M and K were 99.80±0.40 and 99.81±0.40 before premedication (P=0.90), 99.29±0.62 and 99.33±0.63 after premedication (P=0.72), 96.60±0.73 and 97.67±0.79 preoperatively (P=0.68). Significant tachycardia was observed in group K compared to M (P<0.05), but oxygen saturation and respiratory rate changes showed no significant difference throughout the study. Sialorrhea incidence was significantly higher in group K compared to group M (P=0.02) (table 3).

**Table 1:** Baseline patients’ characteristics

Variable		Number	Mean± SD	P-value
Age (year)	Midazolam	35	3.86±1.21	P<0.05
	Ketamine	36	4±1.17	
Weight (Kg)	Midazolam	35	16.13±2.07	P<0.05
	Ketamine	36	16.63±2.34	
Gender	Midazolam(M/F)	35	49.3	P<0.05
	Ketamine(M/F)	36	50.7	

**Table 2:** A Comparison of variables between groups

	Recovery Time	Sedation Score	Separation Score
Midazolam	27.86± 4.42	3.29 ±0.78	2.51±0.61
Ketamine	38.19± 6.67	3±0.71	2.31±0.52
Pvalue	P=0.01	P=0.17	P=0.09

**Table 3:** Frequency distribution of postoperative complications in two groups of patients

**P-value**	**Midazolam**	**Ketamine**	**Variables**	
0.12	0	3	Yes	Restlessness
	35	33	No	
0.02	0	5	Yes	Sialorrhea
	35	31	No	
0.12	0	3	Yes	Vomiting
	35	33	No	
0.20	5	2	Yes	Nausea
	30	34	No	

## Discussion

In line with previous studies, we also found that IN midazolam and ketamine were safe and effective. Both on the five-point sedation scale and separation the mentioned drugs were equally effective and safe. No significant difference was observed in terms of the hemodynamic parameters after premedication except for systolic and diastolic blood pressure which was significantly higher in group K, definitely constant with the cardiovascular effects of the drug inducing sympathomimetic actions through catecholamine release and central nervous system stimulation. On the whole, hemodynamic parameters were kept in the physiologic range. It was also found that recovery time was significantly longer in group K.

The chosen sedation dosage of study drugs was based on the results of previous studies indicating that 5- 9 mg/kg ketamine (16) and 0.2-1 mg/kg midazolam were the recommended dosage for IN route (3). In this work, similar to the PL Narendra’s study, we also believed that deprivation of children from a preventive option for anxiety before surgery was not ethically accepted, therefore a placebo group was not included (5). Supporting this survey, Garcia- Velasco et al., studied the safety and efficacy of IN midazolam and ketamine as premedications with promising results (17). Khatavkar SS investigated the advantage of a combination of IN midazolam with ketamine over midazolam for pediatric patients sedation, they found out that a combination of IN midazolam with ketamine had better results (18). Our results were inconsistent with those of P. L. Narendra's study which showed that both IN midazolam and ketamine as pediatric premedications were effective with no significant differences in sedation scale while midazolam was associated with fewer side-effects (5). But in contrast to our study, SK Bahetwar found that IN ketamine was superior to midazolam with a significantly higher success rate (19). In this work, we found that midazolam produced fewer side effects. Siallorea was highly significant in the ketamine group which is not consistent with that reported by Weksler *et al*., however comparable with the results of the García-Velasco et al.’s study. The use or withholding of atropine or replacing it with glycopyrrolate should be mentioned. Tachycardia was more common in group K. However no serious adverse effect like respiratory depression was reported in study groups. These findings support previous studies (5). In Narendra P.L et al.’s study, 62% of patients in group K and 30% in group M reported at least one side effect (5). Which was not consistent with us as no significant difference was found in terms of adverse reactions between two groups, only for increased secretions in group K. Searching the literature inconstant results are reported which could be partly described by different methodologies. Studied populations regarding age, type of surgeries, evaluation methods, drug dosage, and timing of administration are not the same among studies. Cultural differences and underlying behavioral problems should be considered as well.

Although this study provided some valuable findings, we believe in some limitations. It was a single-center study with a small sample size and also our cases were restricted to urologic surgeries.

Both IN ketamine and midazolam are effective and safe as premedication for children. Fewer side effects were reported in the midazolam group, with shorter recovery time. Obviously further well-planned trials are welcomed. To find practical results, further well-planned trials with a larger sample size including different procedures are recommended.
